# A Simple Weaning Model Based on Interpretable Machine Learning Algorithm for Patients With Sepsis: A Research of MIMIC-IV and eICU Databases

**DOI:** 10.3389/fmed.2021.814566

**Published:** 2022-01-18

**Authors:** Wanjun Liu, Gan Tao, Yijun Zhang, Wenyan Xiao, Jin Zhang, Yu Liu, Zongqing Lu, Tianfeng Hua, Min Yang

**Affiliations:** ^1^The 2nd Department of Intensive Care Unit, The Second Affiliated Hospital of Anhui Medical University, Hefei, China; ^2^The Laboratory of Cardiopulmonary Resuscitation and Critical Care Medicine, The Second Affiliated Hospital of Anhui Medical University, Hefei, China; ^3^Key Laboratory of Intelligent Computing and Signal Processing, Ministry of Education, Anhui University, Hefei, China

**Keywords:** sepsis, invasive mechanical ventilation, weaning, XGBoost, simple prediction model

## Abstract

**Background:**

Invasive mechanical ventilation plays an important role in the prognosis of patients with sepsis. However, there are, currently, no tools specifically designed to assess weaning from invasive mechanical ventilation in patients with sepsis. The aim of our study was to develop a practical model to predict weaning in patients with sepsis.

**Methods:**

We extracted patient information from the Medical Information Mart for Intensive Care Database-IV (MIMIC-IV) and the eICU Collaborative Research Database (eICU-CRD). Kaplan–Meier curves were plotted to compare the 28-day mortality between patients who successfully weaned and those who failed to wean. Subsequently, MIMIC-IV was divided into a training set and an internal verification set, and the eICU-CRD was designated as the external verification set. We selected the best model to simplify the internal and external validation sets based on the performance of the model.

**Results:**

A total of 5020 and 7081 sepsis patients with invasive mechanical ventilation in MIMIC-IV and eICU-CRD were included, respectively. After matching, weaning was independently associated with 28-day mortality and length of ICU stay (*p* < 0.001 and *p* = 0.002, respectively). After comparison, 35 clinical variables were extracted to build weaning models. XGBoost performed the best discrimination among the models in the internal and external validation sets (AUROC: 0.80 and 0.86, respectively). Finally, a simplified model was developed based on XGBoost, which included only four variables. The simplified model also had good predictive performance (AUROC:0.75 and 0.78 in internal and external validation sets, respectively) and was developed into a web-based tool for further review.

**Conclusions:**

Weaning success is independently related to short-term mortality in patients with sepsis. The simplified model based on the XGBoost algorithm provides good predictive performance and great clinical applicablity for weaning, and a web-based tool was developed for better clinical application.

## Introduction

Difficult weaning or prolonged invasive mechanical ventilation is more common in patients with sepsis (1, 2). Lung susceptibility to ventilatory injury is thought to be increased by sepsis (3, 4), and mechanical ventilation may also lead to the exacerbation of pulmonary infection (5). Prolonged mechanical ventilation can lead to a poor prognosis (6, 7). However, insufficient duration of mechanical ventilation is unfavorable for patients. Weaning in unprepared patients leads to increased mortality and prolonged ICU stay (8). Therefore, the choice of an appropriate weaning time is of great importance.

Previous studies on weaning have evaluated numerous methods on weaning, such as rapid shallow breathing index (RSBI) (9), spontaneous breathing experiment (SBT), and compliance, oxygenation, respiratory rate, and pressure (CROP) index. Nevertheless, weaning factors specific to patients with sepsis are scarce. Unfortunately, the accuracy of these factors in predicting weaning is unsatisfactory (10, 11). Moreover, weaning from mechanical ventilation has also been shown to be related to consciousness, diaphragmatic function, and cardiac function (12–14). Traditional prediction of weaning has several limitations. On the one hand, traditional methods of weaning as a complex process are inadequate for the use of clinical indicators utilization. On the other hand, traditional methods have deficits in predictive performance due to disease-related differences in the target population, as there are no specific target populations.

Given the rapid development of clinical medicine, a refined weaning scheme is needed to meet the demands of clinical development. A simple and reliable weaning program could not only effectively assist clinicians but also improve the patient prognosis, especially in patients with sepsis with ventilator dependence.

In this study, we aimed to develop a reliable model for predicting weaning success in patients with sepsis. To this end, we extracted data within 24 h from patients with sepsis before weaning from a large dataset. Features were selected based on their clinical availability and explained by their importance. In addition, our model was further validated using datasets from various sources.

## Methods

### Data Source

Our study was a retrospective cohort study based on the MIMIC-IV (version 1.0) database. This database contains over 40,000 ICU patients from Beth Israel Deaconess Medical Center between 2008 and 2019. Moreover, we used an independent external validation set called eICU-CRD Collaborative Research (eICU-CRD) Database (version 2.0), which is a multicenter database of over 200,000 ICU admissions in the United States. We carefully studied the courses and obtained permission to use the database (record ID 39691989). Because the patient privacy information was encrypted in the database, the ethics committee at the two medical centers did not require informed consent.

### Patients and Definitions

**Figure 1:**
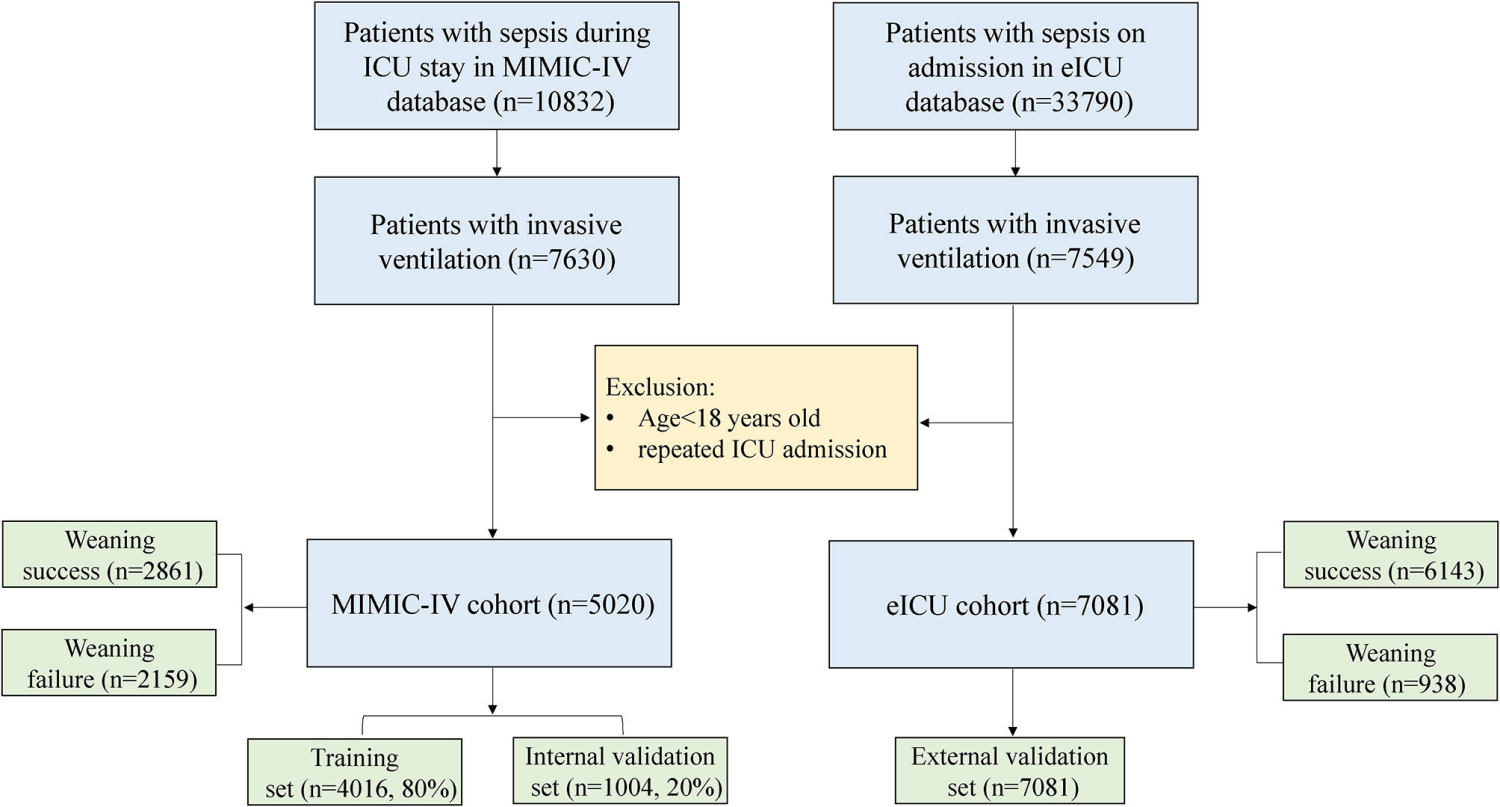
The flow chart of data extraction. eICU-CRD, eICU-CRD Collaborative Research Database. MIMIC-IV, Medical Information Mart for Intensive Care-IV; ICU, intensive care unit.

In this study, sepsis was diagnosed based on the Sepsis-3 criteria [(15); SOFA score ≥ 2, and suspicious infection]. The contents of a recent guideline had been considered before implementing the criteria for successful weaning (16). The definition of weaning success (WS) was as follows: (a) no intubation or invasive ventilation within 48 h after weaning, (b) no death within 48 h after weaning, and (c) noninvasive ventilation time was shorter than 48 h after weaning. The patients who experienced invasive mechanical ventilation and met the criteria for Sepsis-3 were included in both datasets. The exclusion criteria were as follows: (a) repeated ICU admissions and (b) age <18 years (Figure 1).

### Data Collection and Variable Extraction

For modeling, we preferred variables with a single measurement. Clinical importance and shapley additive explanations (SHAP) values were used to further reduce the amount of data. Patient demographics were collected, including age, sex, and body mass index (BMI). Clinical and chemical variables had been extracted within 24 h prior to weaning to create models. The extracted variables were the worst value of the day, which was as follows: arterial blood gas [pH value, arterial oxygen partial pressure (PaO_2_), arterial carbon dioxide partial pressure (PaCO_2_), base excess (BE)], full blood count [white blood cell count (WBC), hemoglobin (HB), platelet (PLT)], laboratory index (creatinine, anion gap), vital signs [heart rate, respiratory rate, mean arterial pressure (MAP), peripheral oxygen saturation (SPO_2_), temperature, oxygenation index (OI)], and urine output. In addition, data on therapeutic measures [days of invasive ventilation, days of antibiotic use, days of continuous renal replacement therapy (CRRT), and vasopressor therapy within 24 h]. The variables in the matched data were from the first day of ICU admission and included the variables listed above (Supplementary Table 1). To further balance the differences in baseline data between the patients with weaning failure (WF) and patients with weaning success, comprehensive indicators were extracted, such as the sequential organ failure assessment (SOFA) score, Glasgow coma scale (GCS) score, and simplified acute physiology score (SAPS II). Comorbidities, as well as infection classification (Supplementary Table 4), were also considered based on the recorded International Classification of Diseases codes (ICD-9 and ICD-10), and the Charlson comorbidity index was also calculated.

### Data Analysis

Continuous variables are described as median and interquartile range (IQR). The Mann–Whitney U-test was used for statistical comparison between the two groups. Categorical variables were described as total number and percentage, and the chi-square test or Fisher's exact test was used for comparison between groups. Propensity score matching (PSM) was used to balance the differences between successful and unsuccessful weaning groups. Inverse probability weighting (IPW) (17) was used to further adjust for possible imbalances between the variables of the two groups. The Kaplan–Meier (K–M) curve was used to describe the 28-day survival rate between the two groups, and the differences in survival rates between groups were compared using the log-rank test.

After comparison, we divided the MIMIC-IV data into two parts: 80% as a training set and 20% as an internal validation set, and the integrated machine-learning algorithm eXtremely Gradient Boosting (XGBoost) to construct a weaning prediction model, which is based on multiple decision trees with gradient boost as a learning framework. The hyperparameters were optimized using a grid search (Supplementary Table 3). Other models, such as KNearest Neighbor (KNN), Multi-Layer Perceptron (MLP), Random Forest (RF), Support Vector Machine (SVM), and Logistic Regression (LR), were also derived from the training set and applied to the test set. The prediction efficiency of the models was compared using a receiver operating characteristic (ROC) curve. In addition, the model was further explained by the SHAP value, demonstrating a linear relationship through local weighted regression scatter smoothing (LOWESS).

Variables with more than 50% missing data were excluded. The missing features of the matched data and model data are shown in Supplementary Figure 1A and Supplementary Table 2. Missing values were input using the multiple imputation method. Due to the different missing datasets, we had extracted only the CROP and RSBI 24 h before weaning in MIMIC-IV, and the predictive performance was also compared with XGBoost in the internal validation set. In addition, the model was further simplified using the recursive feature elimination algorithm (RFE).

Structured query language was used to extract the data from these two databases. All statistical analyses were performed using R 3.6.2 (Chicago, Illinois) and Python (version 3.6.6), and statistical significance was set at *p* < 0.05.

## Results

### Matching Baseline and Clinical Outcomes

**Figure 2:**
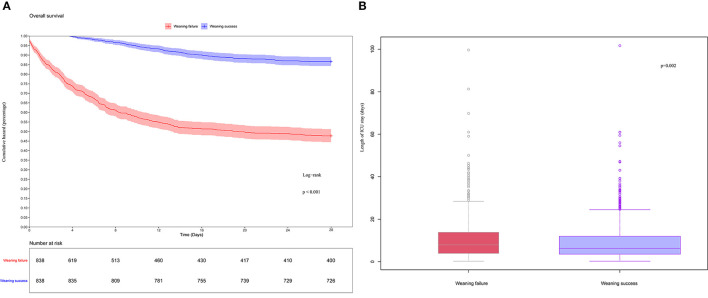
**(A)** K-M curves estimated 28-day survival probability of weaning failure and weaning success patients. **(B)** Box-plot of weaning failure and weaning success patients.

As shown in Figure 1, 5,020 patients with sepsis who received invasive mechanical ventilation were ultimately included in the MIMIC-IV database. The baseline characteristics on the first day of ICU admission are shown in Supplementary Table 1. PSM and IPW were used to better balance the differences between the two groups. A total of 1,676 patients were included (Supplementary Table 1), and the standardized mean difference (SMD) between groups was significantly reduced (Supplementary Figure 1B). In comparison, there was a significant difference in the length of stay in the ICU between the two groups (Figure 2B, *p* = 0.002). Similarly, the WS group had a significantly lower 28-day mortality rate (Figure 2A, *p* < 0.001).

### Baseline Characteristics of Models

In the MIMIC-IV cohort, successful weaning was associated with reductions in highest WBC, highest creatinine, highest anion gap, highest heart rate, highest respiratory rate, highest body temperature, highest PEEP level, antibiotic duration, invasive mechanical ventilation (IMV) duration, and vasopressor use 1 day before weaning (Table 1). Consequently, the performance of these indicators behaved similarly in the eICU-CRD cohort, except for age and the highest FiO_2_ (Table 1). Regarding comorbidity, it was observed that successful weaning benefited chronic pulmonary disease, congestive heart failure, renal disease, and diabetes in the MIMIC-IV cohort. However, except for severe liver disease, other comorbidities were inconsistent between the WS and WF groups (Table 1).

**Table 1:** Baseline characteristics of the MIMIC-IV and eICU cohorts.

**Variables**	**MIMIC-IV cohort**				**eICU cohort**			
	**Overall** **(***n*** = 5,020)**	**Weaning failure** **(***n*** = 2,159)**	**Weaning success** **(***n*** = 2,861)**	***p***	**Overall** **(***n*** = 7,081)**	**Weaning failure** **(***n*** = 938)**	**Weaning success** **(***n*** = 6,143)**	***p***
*n*	5,020	2,159	2,861		7,081	938	6,143	
Age (years)	68 (57, 78)	68 (56, 79)	68 (57, 78)	0.335	64 (53, 74)	68 (57, 77)	63 (52, 73)	<0.001
Male	2,944 (58.6)	1,241 (57.5)	1,703 (59.5)	0.154	3,976 (56.2)	511 (54.5)	3,465 (56.4)	0.283
BMI (kg/m^2^)	28 (24, 33)	28 (24, 33)	28 (24, 32)	0.428	28 (23, 33)	27 (23, 33)	28 (23, 33)	0.02
Chronic pulmonary disease (*n*, %)	1,719 (34.2)	689 (31.9)	1,030 (36.0)	0.003	1,334 (18.8)	175 (18.7)	1,159 (18.9)	0.914
Congestive heart failure (*n*, %)	2,155 (42.9)	788 (36.5)	1,367 (47.8)	<0.001	1,174 (16.6)	158 (16.8)	1,016 (16.5)	0.852
Dementia (*n*, %)	239 (4.8)	88 (4.1)	151 (5.3)	0.056	207 (2.9)	40 (4.3)	167 (2.7)	0.012
Severe liver disease (*n*, %)	547 (10.9)	286 (13.2)	261 (9.1)	<0.001	209 (3.0)	60 (6.4)	149 (2.4)	<0.001
Renal disease (*n*, %)	1,463 (29.1)	574 (26.6)	889 (31.1)	0.001	949 (13.4)	151 (16.1)	798 (13.0)	0.011
Rheumatic disease (*n*, %)	211 (4.2)	84 (3.9)	127 (4.4)	0.375	165 (2.3)	21 (2.2)	144 (2.3)	0.934
Diabetes (%)	1,665 (33.2)	675 (31.3)	990 (34.6)	0.014	2,129 (30.1)	283 (30.2)	1,846 (30.1)	0.971
Charlson comorbidity index	6 (4, 8)	6 (4, 8)	6 (4, 8)	0.082	3 (2, 5)	4 (3, 6)	3.00 (2, 5)	<0.001
GCS	14 (10, 15)	13 (7, 15)	14 (10, 15)	<0.001	8 (6, 10)	4 (3, 8)	9 (6, 10)	<0.001
Highest WBC (×10^9^/L)	14.1 (9.8, 19.9)	14.7 (9.8, 21.3)	13.8 (9.9, 18.9)	<0.001	11.9 (8.7, 16.4)	14.7 (9.7, 20.5)	11.7 (8.6, 15.8)	<0.001
Lowest hemoglobin (g/L)	9.2 (8.0, 10.6)	9.1 (7.9, 10.6)	9.3 (8.1, 10.6)	0.002	9.7 (8.4, 11.3)	9.3 (7.9, 10.8)	9.8 (8.5, 11.4)	<0.001
Lowest platelets (×10^9^/L)	143 (89, 213)	136 (75, 212)	147 (100, 215)	<0.001	167 (113, 233)	141 (76, 212)	170 (118, 236)	<0.001
Highest creatinine (mg/dL)	1.3 (0.9, 2.3)	1.6 (1.0, 2.7)	1.2 (0.8, 1.9)	<0.001	1.1 (0.7, 1.8)	1.7 (1.0, 3.0)	1.0 (0.7, 1.6)	<0.001
Highest anion gap (mEq/L)	15.0 (13.0, 19.0)	17.0 (14.0, 22.0)	14.0 (12.0, 17.0)	<0.001	10.0 (8.0, 14.0)	13.0 (10.0, 18.0)	10.0 (7.7, 13.0)	<0.001
Lowest pH level	7.3 (7.3, 7.4)	7.3 (7.2, 7.4)	7.3 (7.3, 7.4)	<0.001	7.4 (7.3, 7.4)	7.3 (7.2, 7.4)	7.4 (7.3, 7.4)	<0.001
Lowest PaO_2_ (mmHg)	80 (53, 106)	75 (48, 99)	84 (60, 110)	<0.001	85 (69, 115)	78.00 (61, 102)	86 (69, 117)	<0.001
Highest PaCO_2_ (mmHg)	45 (39, 51)	44 (38, 52)	45 (40, 50)	0.837	43 (37, 49)	42 (36, 52)	43 (37, 49)	0.977
Lowest base excess (mEq/L)	−3.0 (−7.0, 0.0)	−4.0 (−10.0, 0.0)	−2.0 (−5.0, 0.0)	<0.001	−1.0 (−5.4, 2.0)	−5.5 (−12.4,0.6)	−0.6 (−5.0, 2.2)	<0.001
Highest heart rate (/min)	103 (90, 119)	108 (94, 124)	100 (88, 115)	<0.001	103 (90, 118)	112 (97, 129)	102.00 (89, 116)	<0.001
Highest respiratory rate (/min)	27 (23, 31)	29 (24, 33)	26 (22, 30)	<0.001	25 (21, 31)	30.00 (25, 35)	25.00 (21, 30)	<0.001
Lowest MAP (mmHg)	60 (54, 65)	59 (52, 64)	60 (56, 66)	<0.001	65 (57, 73)	59 (49, 68)	66 (59, 74)	<0.001
Highest body temperature (°C)	37.4 (37.0, 38.1)	37.5 (36.9, 38.2)	37.4 (37.1, 37.9)	0.021	37.4 (37.0, 37.9)	37.4 (36.9, 38.1)	37.4 (37.1, 37.9)	0.362
Lowest SPO_2_	94 (91, 96)	93 (89, 95)	94 (92, 97)	<0.001	94 (91, 97)	91 (82, 94)	94 (91, 97)	<0.001
Highest PEEP (cmH_2_O)	7 (5, 10)	9 (5, 12)	6 (5, 10)	<0.001	5 (5, 8)	5 (5, 10)	5.00 (5, 6)	<0.001
Lowest tidal volume (ml)	397 (328, 455)	395 (325 452)	398 (329 459)	0.154	422 (343, 497)	423.50 (356, 493)	422.00 (340, 498)	0.591
Lowest OI	174 (105, 250)	152 (91, 232)	188 (120, 260)	<0.001	206 (144, 282)	161 (98, 227)	212 (150, 288)	<0.001
Highest FiO_2_ (%)	50 (40, 80)	50 (40, 90)	50 (40, 80)	<0.001	50 (40, 100)	60 (40, 100)	50 (40, 80)	<0.001
Antibiotic duration (day)	1 (1, 4)	2 (1, 5)	1 (1, 3)	<0.001	0 (0, 2)	1 (0, 4)	0 (0, 2)	<0.001
CRRT duration (day)	0 (0, 0)	0 (0, 0)	0 (0, 0)	<0.001	0 (0, 0)	0 (0, 0)	0 (0, 0)	<0.001
IMV duration (day)	1.5 (0.6, 3.7)	1.9 (0.7, 4.4)	1.2 (0.6, 3.2)	<0.001	2.0 (1.0, 5.0)	3.0 (2.0, 6.0)	2.0 (1.0, 5.0)	<0.001
Urine output (ml/kg/h)	0.6 (0.2, 1.2)	0.5 (0.1, 1.1)	0.7 (0.4, 1.3)	<0.001	0.6 (0.3, 1.1)	0.3 (0.1, 0.8)	0.64 (0.3, 1.1)	<0.001
Vasopressor used 1 day before weaning (*n*, %)	3,882 (77.3)	1,712 (79.3)	2,170 (75.8)	0.004	2,337 (33.0)	511 (54.5)	1,826 (29.7)	<0.001

*Values are presented as median and interquartile range (IQR); BMI, body mass index; GCS, Glasgow coma scale; WBC, white blood cell count; PaO_2_, arterial oxygen partial pressure; PaCO_2_, arterial carbon dioxide partial pressure; MAP, mean arterial pressure; SPO_2_, pulse oxygen saturation; PEEP, positive end expiratory pressure; OI, oxygenation index; FIO_2_, fraction inspired oxygen concentration; CRRT, continuous renal replacement therapy; IMV, invasive mechanical ventilation*.

### Comparison and Explanation of Models

**Figure 3:**
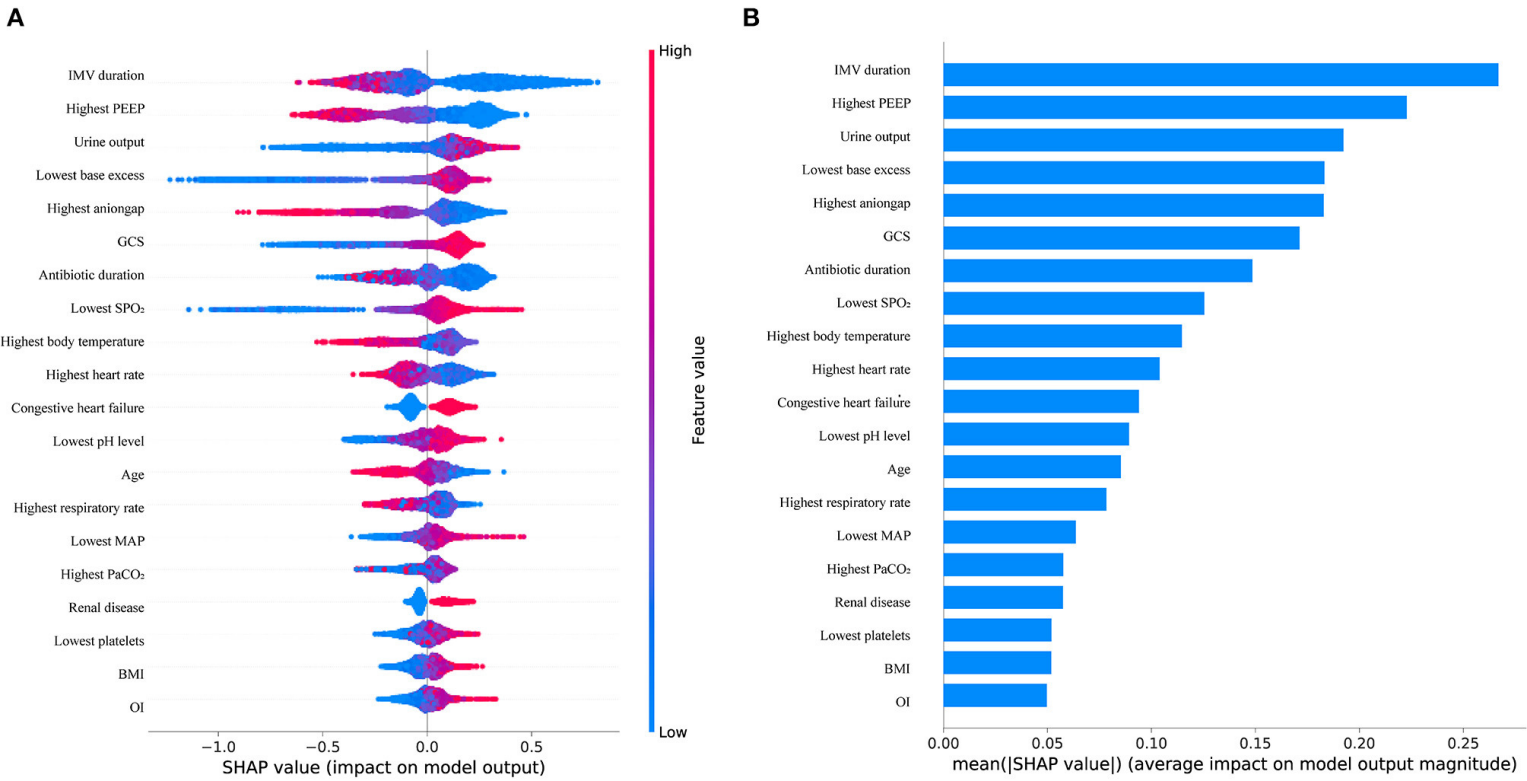
**(A)** Distribution of the impacts of each variable on the output of the XGBoost model estimated using the SHAP values. **(B)** Ranking of variables importance.

**Figure 4:**
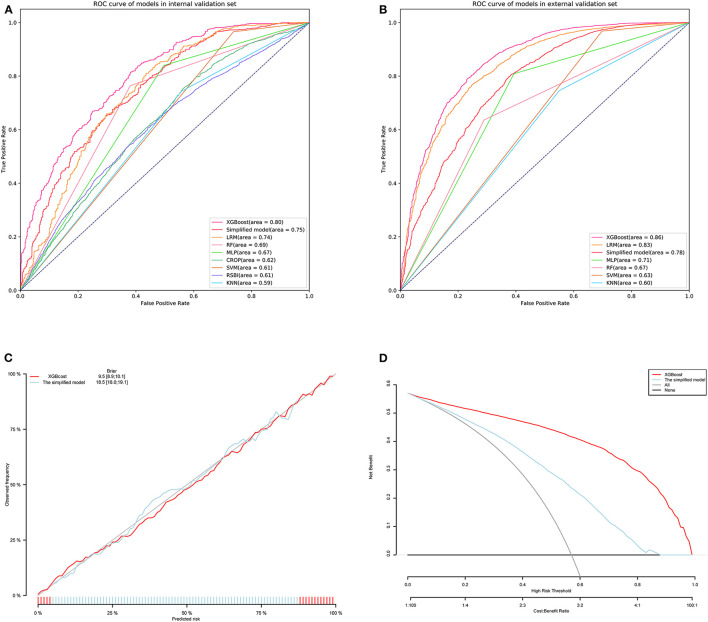
Receiver operating characteristic curves (ROCs) of the XGBoost, LRM, RF, MLP, SVM, KNN, and simplified model. **(A)** Internal validation set. **(B)** External validation set. **(C)** Decision curve analysis of the XGBoost and simplified model. **(D)** Calibration curve of the XGBoost and simplified model. XGBoost, eXtremely gradient boosting; KNN, KNearest neighbor; MLP, multi-layer perceptron; RF, random forest; SVM, support vector machine; LRM, logistic regression; RSBI, rapid shallow breathing Index; CROP, compliance, oxygenation, respiratory rate, pressure index.

We trained the models using the training set from MIMIC-IV. As shown in Table 2, the XGBoost model with all available variables had a striking AUROC of 0.80 [95% confidence interval (CI): 0.77–0.82 in the internal validation set, and 0.86 (95% CI: 0.85–0.87)] in the external validation set, while the other five representative models had the highest AUROC, 00.74 (95% CI: 0.71–0.77) in the internal validation set, and 0.83 (95% CI: 0.82–0.84) in the external validation set. The final hyperparameter settings for XGBoost are listed in Supplementary Table 3. The SHAP values for the XGBoost model were assessed and are shown in Figure 3A. The importance of the variables was sorted by the gap value and is shown in Figure 3B. Figures 4A,B show the comparison between the XGBoost model and the other five models or predictive factors. As can be seen, the XGBoost model significantly outperformed the other five models or predictive factors in both the internal validation and external validation. Due to the extensive missing data, we did not show the ROC curves of CROP and RSBI in the external validation set. In addition, we performed a decision curve analysis (Figure 4C) and a calibration plot (Figure 4D) to illustrate the performance of the XGBoost model.

**Table 2:** The predictive performance of models in the internal and external validation sets.

**Models**	**Internal validation set**						**External validation set**					
	**AUROC**	**Cut-off**	**Sensitivity (%)**	**Specificity (%)**	**PPV (%)**	**NPV (%)**	**AUROC**	**Cut-off**	**Sensitivity (%)**	**Specificity (%)**	**PPV (%)**	**NPV (%)**
XGBoost	0.80 (0.77–0.82)	0.57	0.75 (0.71–0.78)	0.71 (0.66–0.75)	0.81 (0.79–0.84)	0.62 (0.58–0.65)	0.86 (0.85–0.87)	0.51	0.96 (0.95–0.96)	0.36 (0.34–0.38)	0.79 (0.79–0.80)	0.77 (0.74–0.79)
The simplified model	0.75 (0.72–0.77)	0.58	0.74 (0.70–0.78)	0.61 (0.56–0.65)	0.68 (0.65–0.70)	0.68 (0.64–0.72)	0.78 (0.77–0.79)	0.54	0.93 (0.93–0.94)	0.32 (0.30–0.34)	0.80 (0.79–0.80)	0.63 (0.60–0.65)
MLP	0.67 (0.64–0.74)	1	0.84 (0.81–0.87)	0.50 (0.46–0.55)	0.84 (0.81–0.86)	0.50 (0.47–0.54)	0.71 (0.69–0.72)	1	0.93 (0.92–0.94)	0.33 (0.30–0.35)	0.81 (0.80–0.81)	0.61 (0.58–0.63)
RF	0.69 (0.66–0.72)	1	0.73 (0.69–0.77)	0.66 (0.61–0.70)	0.76 (0.74–0.79)	0.62 (0.58–0.65)	0.67 (0.66–0.68)	1	0.93 (0.93–0.94)	0.23 (0.21–0.25)	0.64 (0.63–0.64)	0.71 (0.68–0.74)
SVM	0.61 (0.58–0.64)	1	0.64 (0.61–0.67)	0.85 (0.77–0.90)	0.97 (0.95–0.98)	0.26 (0.24–0.29)	0.63 (0.62–0.65)	1	0.90 (0.89–0.91)	0.59 (0.55–0.64)	0.97 (0.96–0.97)	0.30 (0.28–0.32)
LR	0.74 (0.71–0.77)	0.55	0.72 (0.68–0.75)	0.68 (0.63–0.73)	0.81 (0.78–0.83)	0.57 (0.53–0.59)	0.83 (0.82–0.84)	0.58	0.95 (0.94–0.96)	0.33 (0.31–0.35)	0.77 (0.76–0.77)	0.75 (0.73–0.78)
KNN	0.59 (0.56–0.62)	1	0.65 (0.61–0.68)	0.56 (0.50–0.61)	0.74 (0.72–0.77)	0.44 (0.41–0.48)	0.59 (0.58–0.61)	1	0.89 (0.89–0.91)	0.21 (0.19–0.23)	0.75 (0.74–0.75)	0.45 (0.42–0.48)

*XGBoost, eXtremely gradient boosting; MLP, multilayer perceptron; RF, random forest; SVM, support vector machine; LR, logistic regression; KNN, KNearest neighbor; AUROC, area under the receiver operating characteristic curves; PPV, positive predictive value; NPV, negative predictive value*.

### SHAP Values Depending on Variables

**Figure 5:**
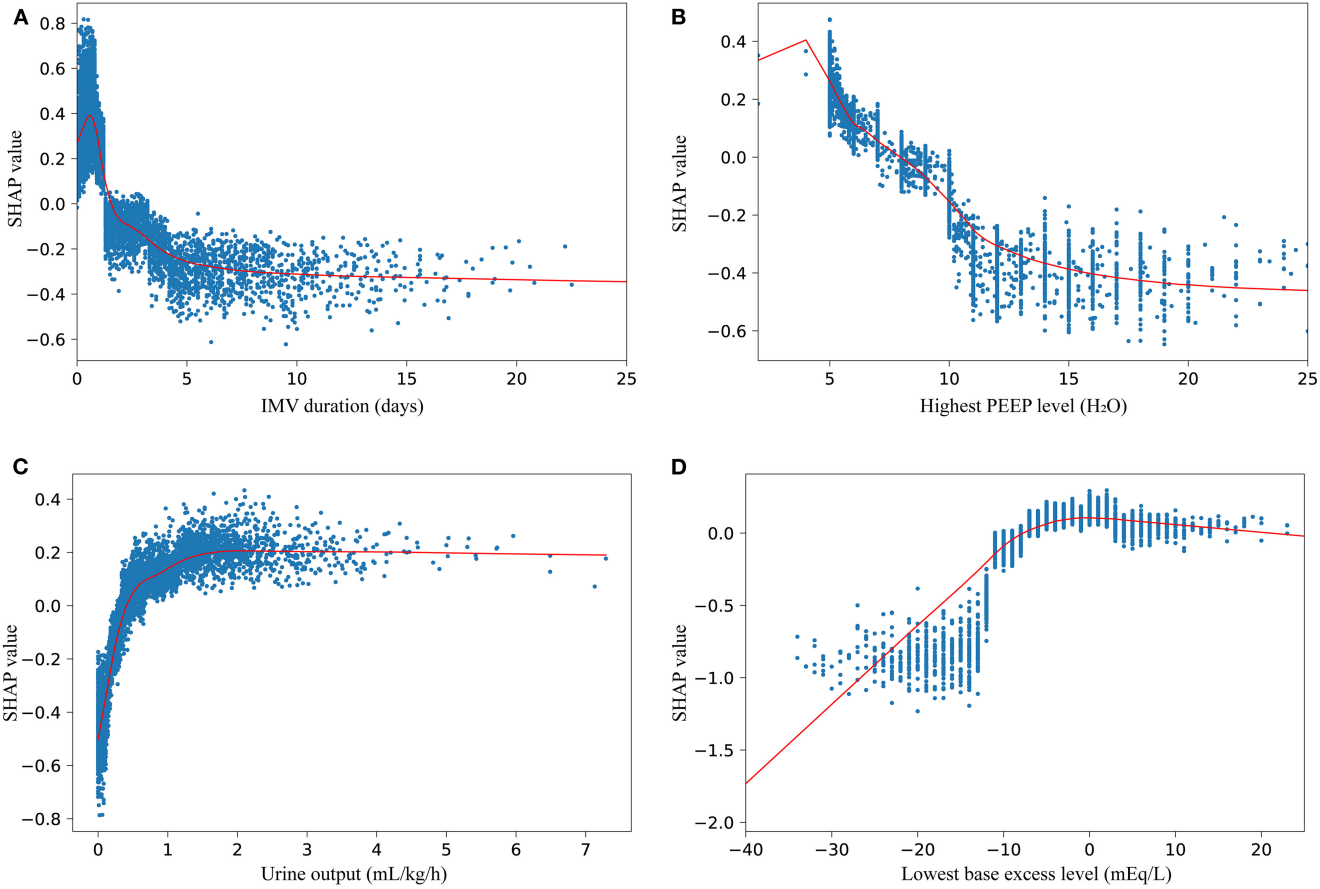
SHAP dependency plots of IMV duration **(A)**, highest PEEP level **(B)**, urine output **(C)**, and lowest base excess level **(D)**.

The probability of successful weaning increases with an increase in the following indicators: urine output, lowest base excess, GCS, lowest SPO_2_, congestive heart failure, lowest pH, lowest map, highest PaCO_2_, renal disease, lowest platelet count, BMI, and OI (Figure 3A). The contribution of each feature in the internal validation set is shown in Figure 3B in order of importance. Finally, we performed a partial dependency plot of the four contributing continuous variables to explain the impact of the change in value of each variable on the patients with WS, as shown in Figure 5. The remaining variables are shown in Supplementary Figures 2, 3. As shown in the partial dependency plot, feature values are indicated by a blue scatter plot, with the linear relationship represented by a red curve, where SHAP values represent an increase in the probability of WS when the value is positive and *vice versa*.

Figure 5 shows the change trend of WS probability with the change in variables. For some variables, there is a discernable trend where the WS probability increases with an increase in the value of variables. These variables are urine output (Figure 5C), lowest BE (Figure 5D), GCS (Supplementary Figure 2B), and lowest SPO_2_ (Supplementary Figure 2D). In contrast, the decrease in some indicators, such as the highest PEEP level (Figure 5B), the highest anion gap level (Supplementary Figure 2A), and age (Supplementary Figure 2H), suggests a decrease in WS probability. Additionally, some variables seem to have a reasonable value, and the best cut-off value for these variables can be roughly judged by the LOWESS curve (Figure 5A; Supplementary Figures 2C,E–G). To obtain the best WS possibility, the values of these variables should be kept close to the cut-off value.

### The Web-Based Tool and an Example Scenario

We simplified the previous XGBoost model according to the variable importance. The four variables (IMV duration, highest PEEP level, urine output, and lowest base excess) with the highest importance were used to develop a simplified model; the performance of the simplified model is shown in Figure 4 and Table 2. As shown, although the predictive performance of the simplified model decreased slightly, the model was greatly simplified. Subsequently, a web-based tool was developed for clinicians to use a simplified model. The tool can be accessed at http://49.235.211.121/frontdoc/sepweaning.html. After submitting the required data, the probability of weaning was calculated based on the simplified model.

When patients with sepsis are ready to be weaned from invasive mechanical ventilation, clinicians can make a quick decision using the SHAP dependency plots. For scenarios that require precise calculation, the web tool can provide an accurate probability of successful weaning based on the input indicators.

## Discussion

This retrospective analysis included two large study cohorts from MIMIC-IV and eICU-CRD. First, we compared mortality and length of ICU stay from WS and WF in patients with sepsis using an effective balance method. Unfortunately, we found significant differences in mortality between patients with WS and WF. Therefore, the XGBoost model and the other five models were applied and evaluated sequentially to identify the beneficial factors associated with WS of patients with sepsis in the ICU. To our knowledge, this is the first study to predict weaning in patients with sepsis based on extensive public data. The difference from previous studies is that we developed an integrated machine learning model with high performance. In addition, we fully evaluated our model using another equally large public dataset. Finally, we explained the main variables and described the effects of their changing trends on weaning.

The matched results showed that there was a significant difference in the length of stay in the ICU between the WS and WF groups (Figure 1B, *p* = 0.002). This trend was not confirmed in the external validation set (Supplementary Figure 4C). Nevertheless, successful weaning of patients with sepsis significantly reduced mortality (Figure 1A, *p* < 0.001), and the matched result from the external validation set showed a similar trend (Supplementary Figure 4B). These results were comparable to those of previous studies (18). Therefore, successful weaning is a key factor in improving survival and potentially shortening ICU stay.

In both the internal validation set and the external validation set, our model showed excellent reliability and prospective generalization ability, and the prediction performance was significantly better than that of the traditional method (Figure 4C). In the model, the contribution to the prediction of WS varied with the variables. Previous studies have shown that the duration of IMV plays an important role in weaning (19–21). However, because IMV duration is the strongest variable in our model, the contribution of IMV duration to these models is inconsistent. We only included patients with sepsis admitted to the ICU in our study. Therefore, population diversity could lead to this difference. Moreover, it was observed that the best IMV duration was maintained for about 1 day according to the LOWESS curve (Figure 5A). Other variables with prospective cut-off values were antibiotic duration (Supplementary Figure 2C), highest body temperature (Supplementary Figure 2E), highest heart rate (Supplementary Figure 2F), highest respiratory rate (Supplementary Figure 3A), and highest PaCO_2_ (Supplementary Figure 3C). Although these variables have also been mentioned in previous studies (19, 21, 22), the trends and specific contributions of these variables have not been clarified.

In our study, age showed a clear downward trend of successful weaning ability with increasing value, especially in patients aged >75 years (Supplementary Figure 2H). This trend is supported by increasing evidence (19, 23). Interestingly, BMI and congestive heart failure as low-contributing variables were proportional to WS probability in our study. This finding is supported by previous studies (24, 25). However, there is also an opposite conclusion (19). In a study on viral infections, excessive BMI may lead to worse clinical outcomes (26). However, in another study, even a reasonably high BMI can help patients improve their disease (27). In summary, these differences may be due to the diversity of the population and the diversity of diseases.

PEEP, urine output, and SPO_2_, as the most commonly measured indices, play a key role in predicting WS. In this study, a low PEEP strategy was found to be more beneficial for patients with sepsis weaning from ventilation. Although the low-level PEEP strategy did not have a significant effect on improving ventilator weaning in a cohort study (28), it still has the potential to promote ventilator weaning (29). There is increasing evidence that high levels of PEEP are a risk factor in reducing the likelihood of WS (19, 30). As we have considered, high levels of PEEP lead to lung congestion and increase the respiratory burden of patients (31), which may explain why high levels of PEEP play a positive role in the weaning process (Figure 5B). Urinary output is the primary means for humans to maintain fluid balance, and excellent fluid management strategies could significantly improve patient survival (32). Polyuria has been shown to have no negative impact on weaning (33), but negative fluid balance significantly impedes weaning success (34, 35). These findings are consistent with those of the present study (Figure 5C). The pH was an interesting finding in our study. As the most important indicator of acid-base balance, an abnormally high or low pH had a detrimental effect on weaning in our study. Nevertheless, low pH was more likely to cause weaning failure (Supplementary Figure 2G). This finding is consistent with previous studies, as low pH of extracellular fluid compromises the immune function of the body in patients with sepsis (36). BE and PaCO_2_ are indicators closely related to pH. Early studies have shown that removing PaCO_2_ from the body effectively improves the success rate of weaning (37, 38). The relationship between BE and weaning has not yet been studied. In any case, extracellular acid-base balance is susceptible to the interaction of several variables. For the three variables of BE, PaCO_2_, and pH, we used an interactive effects plot to explain the relationship between the three variables. As can be seen in Supplementary Figure 3, the tendency of these three variables to predict weaning tended to be consistent, which also shows the rationality of using this model.

In clinical practice, weaning should be a medical behavior that needs careful consideration. Inappropriate weaning may lead to worsening of disease, higher mortality, longer hospital stays, and higher hospital costs (7, 39, 40). Therefore, a simple and effective prediction method is needed. Compared with the traditional prediction model, our model has better prediction performance (11, 41). Although some new weaning models have been proposed in recent years (19, 20, 42, 43), the performance of the model varies according to the target population. In patients with sepsis, the developed model was able to predict weaning well, as reflected by a high AUROC value of 0.80 and 0.86 in the internal and external validation sets, respectively. Interestingly, the performance of the model was better in patients with pulmonary infections than in the validation sets (Supplementary Figure 5). Obviously, the performance of our model was better in the external validation set. Apart from the good generalization ability of our model, we believe that the different data sources could explain this phenomenon, which also occurs in other studies (44). Finally, we simplified our model and developed a web-based tool that allows convenient use of the model.

There are still potential limitations in our research. First, because of the limitation of the database, other variables that could have predictive value, such as lactate and central venous pressure, with excessively high error rates, were not included in the model. Considering the availability of comprehensive clinical indicators, such as SOFA and SAPS, were not included in the model, although these indicators could improve the predictive performance of the model (19, 45, 46). Second, although our model was validated using data from multiple data sources, we still need additional data sources to further demonstrate the generalizability of the model. Third, in our study, the positive predictive value (PPV) and negative predictive value (NPV) were 0.81, 0.62, 0.79, and 0.77 in the internal and external validation sets, respectively, which means that the model still has some degree of false-positive and false-negative rates. More valuable variables and dynamic prediction models could improve the performance.

In conclusion, weaning success is independent of short-term mortality in patients with sepsis. We developed a prospective model for weaning from invasive mechanical ventilation using the XGBoost algorithm. This model included 35 conventional clinical variables and proved to be more interpretable and predictive. In addition, the model was simplified, and a web-based tool was developed for better clinical application.

## Data Availability Statement

Publicly available datasets were analyzed in this study. This data can be found here: https://physionet.org/about/database/.

## Author Contributions

MY and WL carried out the concepts, design, data acquisition, and manuscript preparation. GT, ZL, and YZ carried out literature search and manuscript preparation. WX, JZ, YL, and TH performed manuscript review, including revision of key technical content and English expression. All authors have read and approved the content of the manuscript.

## Funding

This work is supported by National Natural Science Foundation of China (Grant No. 82072134).

## Conflict of Interest

The authors declare that the research was conducted in the absence of any commercial or financial relationships that could be construed as a potential conflict of interest.

## Publisher's Note

All claims expressed in this article are solely those of the authors and do not necessarily represent those of their affiliated organizations, or those of the publisher, the editors and the reviewers. Any product that may be evaluated in this article, or claim that may be made by its manufacturer, is not guaranteed or endorsed by the publisher.
