# Supplementary material for: A Simple Weaning Model Based on Interpretable Machine Learning Algorithm for Patients With Sepsis: A Research of MIMIC-IV and eICU Databases
Source: Front Med (Lausanne). 2022 Jan 18;8:814566. doi: 10.3389/fmed.2021.814566 (PMC8804204; doi:10.3389/fmed.2021.814566)
Supplement: Supplementary file 1 [file Table_1.DOCX]

**Supplementary** **Figure 1**. The matched results in MIMIC-IV cohort. (a) Missing map of variables before matching; (b) Standardized mean difference of variables before and after matching.

**Supplementary** **Figure 2.** SHAP dependency plots of highest anion gap level(a), GCS (b), antibiotic duration (c), lowest SPO_2_ (d), highest body temperature level (e), highest heart rate (f), lowest PH(g), and age (h).

**Supplementary** **Figure 3**. SHAP dependency plots of highest respiratory rate (a), lowest MAP (b), highest PaCO_2_ (c), lowest platelets (d), BMI(e), lowest OI (f); Interactive SHAP dependency plots of PH-BE (g) and PH-PaCO_2_ (h).

**Supplementary Figure 4.** The matched results in eICU-CRD. (a) Standardized mean difference of variables before and after matching. (b) K-M curves estimated 28-day survival probability of weaning failure and weaning success patients in eICU-CRD. (c) Box-plot of weaning failure and weaning success patients.

**Supplementary Figure 5.** Receiver operating characteristic curves (ROCs) of trained models among different infection classifications.
