# Supplementary material for: A Simple Weaning Model Based on Interpretable Machine Learning Algorithm for Patients With Sepsis: A Research of MIMIC-IV and eICU Databases
Source: Front Med (Lausanne). 2022 Jan 18;8:814566. doi: 10.3389/fmed.2021.814566 (PMC8804204; doi:10.3389/fmed.2021.814566)
Supplement: Supplementary file 2 [file Table_2.DOCX]

Supplementary Table 1. Baseline characteristics before and after matching

| Variables | Before matching | | | | After matching | | | |
| --- | --- | --- | --- | --- | --- | --- | --- | --- |
|  | Overall (n=5020) | Weaning failure (n=2159) | Weaning success (n=2861) | p | Overall (n=1676) | Weaning failure (n=838) | Weaning success (n=838) | p |
| Age (years) | 68[57, 78] | 68[56, 78] | 68[57, 78] | 0.335 | 69[57, 79] | 69[57, 79] | 69[58, 79] | 0.899 |
| Male (n,%) | 2944 (58.6) | 1241 (57.5) | 1703 (59.5) | 0.154 | 1002 (59.8) | 489 (58.4) | 513 (61.2) | 0.252 |
| BMI (n,%) | 28.1[24.2, 33.2] | 28.2[24.1, 33.4] | 28.1[24.1, 32.9] | 0.556 | 28.2[24.2, 33.7] | 28.4[24.0, 33.8] | 27.9[24.3, 33.4] | 0.607 |
| SICU (n,%) | 1127 (22.5) | 527 (24.4) | 600 (21.0) | 0.004 | 419 (25.0) | 199 (23.7) | 220 (26.3) | 0.259 |
| Chronic pulmonary disease (n,%) | 1719 (34.2) | 689 (31.9) | 1030 (36.0) | 0.003 | 557 (33.2) | 278 (33.2) | 279 (33.3) | 1 |
| Congestive heart failure (n,%) | 2155 (42.9) | 788 (36.5) | 1367 (47.8) | <0.001 | 703 (41.9) | 349 (41.6) | 354 (42.2) | 0.843 |
| Dementia (n,%) | 239 (4.8) | 88 (4.1) | 151 (5.3) | 0.056 | 88 (5.3) | 44 (5.3) | 44 (5.3) | 1 |
| Severe liver disease (n,%) | 547 (10.9) | 286 (13.2) | 261 (9.1) | <0.001 | 189 (11.3) | 95 (11.3) | 94 (11.2) | 1 |
| Renal disease (n,%) | 1463 (29.1) | 574 (26.6) | 889 (31.1) | 0.001 | 513 (30.6) | 258 (30.8) | 255 (30.4) | 0.916 |
| Diabetes (n,%) | 1665 (33.2) | 675 (31.3) | 990 (34.6) | 0.014 | 565 (33.7) | 292 (34.8) | 273 (32.6) | 0.352 |
| GCS | 10 [5, 14] | 8[3, 12] | 11[7, 14] | <0.001 | 10[6, 13] | 10[6, 13] | 10[6, 13] | 0.415 |
| Charlson comorbidity index | 6[4, 8] | 6[4, 8] | 6 [4, 8] | 0.082 | 6[4, 8] | 6[4, 8] | 6[4, 8] | 0.803 |
| SAPSII | 48 [38, 59] | 52 [42, 65] | 45 [37, 55] | <0.001 | 48[39, 58] | 49[38, 58] | 47[39, 58] | 0.758 |
| SOFA | 11 [8, 13] | 12[9, 14] | 10[7, 12] | <0.001 | 10[8, 13] | 10[8, 13] | 11[8, 13] | 0.579 |
| MELD | 18[11, 25] | 20[13, 28] | 16[11, 23] | <0.001 | 18[11, 25] | 18[11, 25] | 18[11, 25] | 0.637 |
| Mean MAP (mmHg) | 55 [49, 60] | 54[47, 60] | 56[50, 60] | <0.001 | 55[49, 60] | 55[49, 61] | 56[50, 60] | 0.746 |
| Mean heart rate (/min) | 87[77, 100] | 90 [78, 104] | 84[76, 97] | <0.001 | 87[76, 99] | 88[76, 101] | 86[76, 98] | 0.204 |
| Mean respiratory rate (/min) | 20[17, 23] | 21[18, 24] | 19[17, 22] | <0.001 | 20[18, 23] | 20[17, 23] | 20[18, 23] | 0.767 |
| Highest body temperature (℃) | 37.4[36.9, 38.2] | 37.4[36.9, 38.2] | 37.5[37.1, 38.1] | 0.001 | 37.5[37.1, 38.1] | 37.5[36.9, 38.3] | 37.4[37.1, 38.0] | 0.213 |
| Highest WBC (×10^9^  /L) | 16.4[11.6, 22.5] | 16.6[11.1, 23.1] | 16.3[11.9, 22.1] | 0.953 | 16.3[11.8, 22.0] | 16.1[11.4, 21.7] | 16.6[12.0, 22.1] | 0.084 |
| Lowest hemoglobin level (× 10^12^/L) | 9.4[7.9, 11.1] | 9.4[7.9, 11.2] | 9.4[8.0, 11.0] | 0.434 | 9.6[8.0, 11.3] | 9.5[7.9, 11.2] | 9.8[8.1, 11.4] | 0.068 |
| Lowest platelets (×10^9^/L) | 147[95, 209] | 146[84, 213] | 147[101, 207] | 0.01 | 155[101, 219] | 156[96, 221] | 154[103, 216] | 0.787 |
| Highest PT level (s) | 16.2[13.7, 20.9] | 16.90[13.8, 24.2] | 15.80[13.7, 19.3] | <0.001 | 15.7[13.5, 20.5] | 15.5[13.4, 20.5] | 15.8[13.6, 20.7] | 0.180 |
| Highest INR level | 1.5[1.2, 1.9] | 1.60[1.2, 2.3] | 1.40[1.2, 1.8] | <0.001 | 1.4[1.2, 1.9] | 1.4[1.2, 1.9] | 1.4[1.2, 1.9] | 0.219 |
| Highest lactate level (mmol/L) | 3.2[1.9, 5.8] | 3.8[2.0, 7.5] | 2.9[1.8, 4.8] | <0.001 | 2.9[1.8, 5.2] | 2.8[1.8, 5.2] | 3.0[1.8, 5.2] | 0.666 |
| Lowest PaO_2_ (mmHg) | 59[40, 86] | 53[39, 78] | 65[41, 92] | <0.001 | 58[40, 85] | 58[41, 84] | 59[40, 85] | 0.939 |
| Highest PaCO_2_ (mmHg) | 49[42, 58] | 50[43, 61] | 48[42, 56] | <0.001 | 49[42, 58] | 48[42, 58] | 49[42, 58] | 0.493 |
| Lowest OI | 120[75, 205] | 103[66, 187] | 133[84, 217] | <0.001 | 120[77, 210] | 120[74, 216] | 119[79, 208] | 0.633 |
| Infection classification |  |  |  |  |  |  |  |  |
| Pulmonary (n,%) | 1750 (34.9) | 834 (38.6) | 916 (32.0) | <0.001 | 629 (37.5) | 332 (39.6) | 297 (35.4) | 0.086 |
| Catheter (n,%) | 121 (2.4) | 61 (2.8) | 60 (2.1) | 0.116 | 45 (2.7) | 27 (3.2) | 18 (2.1) | 0.227 |
| Urinary tract (n,%) | 775 (15.4) | 300 (13.9) | 475 (16.6) | 0.01 | 273 (16.3) | 137 (16.3) | 136 (16.2) | 1 |
| Intestinal (n,%) | 276 (5.5) | 128 (5.9) | 148 (5.2) | 0.271 | 105 (6.3) | 57 (6.8) | 48 (5.7) | 0.42 |
| Urine output (mL/kg/h) | 0.61[0.24, 1.17] | 0.46[0.10, 1.06] | 0.70[0.35, 1.25] | <0.001 | 0.62[0.27, 1.17] | 0.64[0.21, 1.25] | 0.60[0.33, 1.11] | 0.461 |

BMI, Body mass index; SICU, Surgery intensive care unit; GCS, Glasgow coma scale; MELD, Model for end-stage liver disease; SAPSII, The simplified acute physiology score II; SOFA, Sequential organ failure assessment; WBC, White blood cell count; PT, Prothrombin time; INR, International normalized ratio; PaO_2_, Arterial oxygen partial pressure; PaCO_2_, Arterial carbon dioxide partial pressure; MAP, Mean arterial pressure.

Supplementary Table 2. Distribution of missing data for model variables

| Variables | MIMIC-IV | eICU |
| --- | --- | --- |
| Age (n,%) | 0(0) | 0(0) |
| Male (n,%) | 0(0) | 0(0) |
| BMI (n,%) | 873(17.4) | 75(1.1) |
| Chronic pulmonary disease (n,%) | 0(0) | 0(0) |
| Congestive heart failure (n,%) | 0(0) | 0(0) |
| Dementia (n,%) | 0(0) | 0(0) |
| Severe liver disease (n,%) | 0(0) | 0(0) |
| Renal disease (n,%) | 0(0) | 0(0) |
| Rheumatic disease (n,%) | 0(0) | 0(0) |
| Diabetes (%) | 0(0) | 0(0) |
| Charlson comorbidity index (n,%) | 0(0) | 0(0) |
| GCS | 1921(38.3) | 1801(25.4) |
| Highest WBC(n,%) | 90(1.8) | 488(6.9) |
| Lowest hemoglobin (n,%) | 89(1.8) | 472(6.7) |
| Lowest platelets (n,%) | 90(1.8) | 533(7.5) |
| Highest creatinine (n,%) | 63(1.3) | 477(6.7) |
| Highest aniongap (n,%) | 92(1.8) | 1724(24.3) |
| Lowest pH level (n,%) | 414(8.2) | 2519(35.6) |
| Lowest PaO_2_ (n,%) | 413(8.2) | 2431(34.3) |
| Highest PaCO_2_ (n,%) | 414(8.2) | 2500(35.3) |
| Lowest base excess (n,%) | 414(8.2) | 3169(44.8) |
| Highest heart rate (n,%) | 2(0) | 201(2.8) |
| Highest respiratory (n,%) | 0(0) | 386(5.5) |
| Lowest MAP (n,%) | 153(3) | 947(13.4) |
| Highest temperature (n,%) | 546(10.9) | 233(3.3) |
| Lowest SPO_2_ (n,%) | 12(0.2) | 1255(17.7) |
| Highest PEEP (n,%) | 2(0) | 492(6.9) |
| Lowest tidal volume (n,%) | 16(0.3) | 1802(25.4) |
| Lowest OI (n,%) | 504(10) | 2924(41.3) |
| Highest FiO_2_ (n,%) | 538(10.7) | 2680(37.8) |
| Antibiotic day (n,%) | 0(0) | 0(0) |
| CRRT day (n,%) | 0(0) | 0(0) |
| Invasive ventilation day (n,%) | 0(0) | 0(0) |
| Urine output (n,%) | 254(5.1) | 2185(30.9) |
| Vasopressor used 1 day before weaning (n,%) | 0(0) | 0(0) |

BMI, Body mass index; GCS, Glasgow coma scale; WBC, White blood cell count; PaO_2_, Arterial oxygen partial pressure; PaCO_2_, Arterial carbon

dioxide partial pressure; MAP, Mean arterial pressure; SPO2, Pulse oxygen saturation; PEEP, Positive end expiratory pressure; OI, Oxygenation index;

FIO_2_, Fraction inspired oxygen concentration; CRRT, Continuous renal replacement therapy; IMV, Invasive mechanical ventilation.

Supplementary Table 3. The final hyperparameter of settings

| Models | MIMIC-IV | eICU | The final settings |
| --- | --- | --- | --- |
| XGBoost | depth | Choice | 7 |
|  | min_child_weight | Choice | 1 |
|  | reg_lambda | Uniform | 15 |
|  | learning_rate | Uniform | 0.01 |
|  | gamma | Uniform | 0.15 |
| MLP | hidden_layer_sizes | Uniform | 50 |
|  | learning_rate_int | Uniform | 0.001 |
|  | alpha | Uniform | 0.0001 |
|  | max_iter | Uniform | 200 |
| RF | n_estimators | Uniform | 200 |
|  | max_features | Choice | 5 |
| SVM | C | Choice | 1 |
|  | gamma | Uniform | 0.01 |
| LR | fit_intercept | bool | True |
|  | normalize | bool | False |
| KNN | n_neighbors | Choice | 10 |
|  | p | Choice | 1 |

XGBoost, eXtremely Gradient Boosting; MLP, Multilayer perceptron; RF, Random forest; SVM, Support vector machine; LR, Logistic regression; KNN, KNearest neighbor.

Supplementary Table 4. The ICD code of infection classification

| Infection classification | ICD version | MIMIC-IV | eICU* |
| --- | --- | --- | --- |
| Pulmonary infection | 9 | 46430,48284,48239,4843,51189,48230,4871,486,46450,5118,4801,  481,8289,4838,4829,4809,46611,48283,46400,4821,4660,4808,5111,  5110,462,4650,48231,4611,46421,4830,4659,48241,463,4658,485,  4802,4848,4618,4613,4820,4800,4846,5119,48249,46411,46619,  48282,48232,4847,4822,48242,4841,4610,4870,5100,48281,4619,  46451,460,46410,4878,46431,5109,51181,48240,4612 | 4847,481,4610,46619,46431,4831,4660,5100,4808,  46430,5109,48231,486,4809,4830,4611,48241,48283,  4612,4619,4822,4613,4846,48284,5119 |
|  | 10 | J151,J09X3,J09X1,J1289,J101,J156,J211,J0190,J0180,J0390,  J0410,J0511,J09X9,J0141,J219,J205,J111,J1100,J120,J102,  J129,J1529,J189,J153,J0120,J00,J123,J201,J154,J0191,J210,  J209,J020,J155,J0411,J188,J17,J13,J122,J042,J158,J181,J15211,  J218,J112,J0300,J1001,J1520,J851,J1008,J157,J1000,J208,J852,  J22,J0380,J14,J869,J0130,J0391,J1089,J0140,J853,J040,J0431,  J150,J204,J168,J182,J1081,J09X2,J180,J0430,J0301,J1108,J028,  J0100,J860,J121,J0510,J850,J0110,J069,J029,J060,J1082,J1189,  J1181,J159,J15212 | - |
| Urinary tract infection | 9 | 0980,09837,09817,5990 | 5990 |
|  | 10 | N99521,N390,A5400,A562 | - |
| Intestinal infection | 9 | 00867,0069,0074,00589,00846,0059,0048,00809,00804,0041,  00843,0088,0062,0040,0043,0093,0020,0078,0091,00841,0071,  0049,0090,0030,00800,00844,0083,00324,00845,0050,0085,  00863,0039,00581,00861,00849,0031,00869,00329,0038,0068,  0029,00847,0092,00862 | 0030,00800,00845,00843,0091,0049,0059 |
|  | 10 | A048,A039,A088,A021,A0811,A030,A0471,A046,A0100,A059,  A0839,A082,A029,A033,A028,A078,A047,A069,A071,A080,  A072,A0222,A0472,A050,A084,A09,A043,A049,A038,A031,  A044,A045,A020 | - |

* The ICD-10 code is not found in the eICU database.
