# Supplementary figures and images for: A Simple Weaning Model Based on Interpretable Machine Learning Algorithm for Patients With Sepsis: A Research of MIMIC-IV and eICU Databases
Source: Front Med (Lausanne). 2022 Jan 18;8:814566. doi: 10.3389/fmed.2021.814566 (PMC8804204; doi:10.3389/fmed.2021.814566)

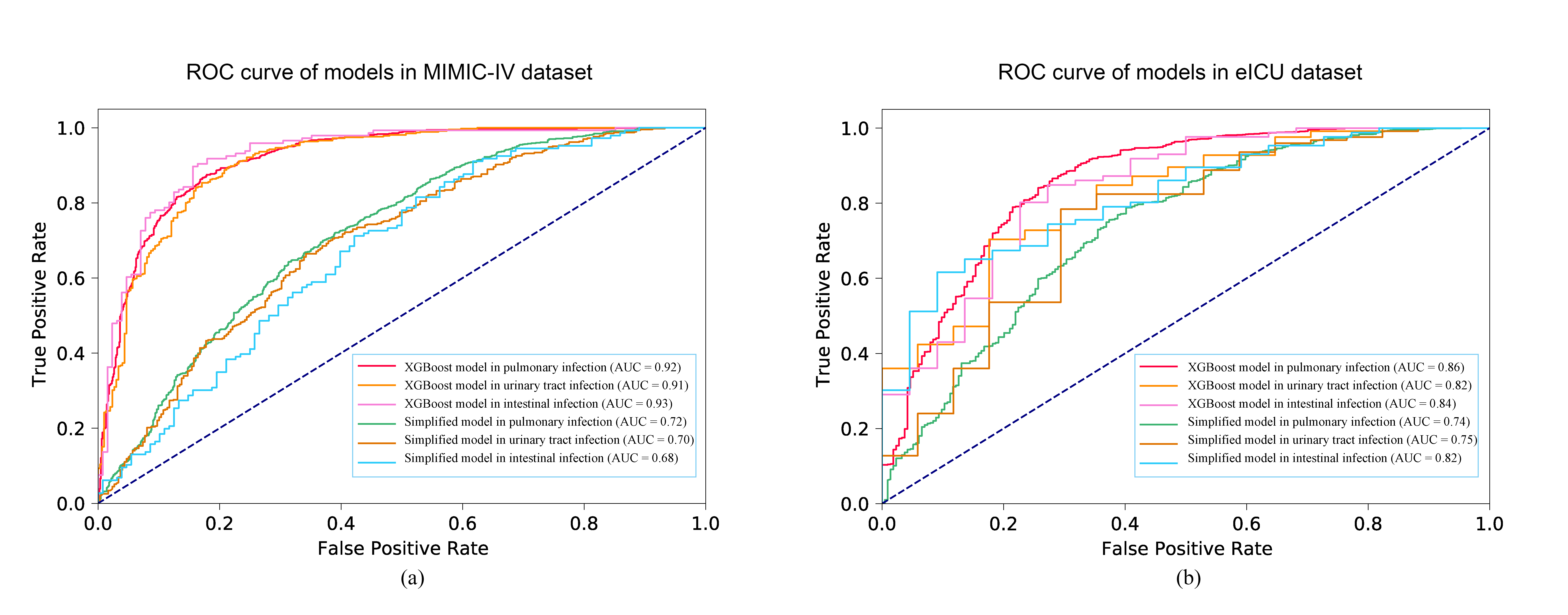

Supplement: Supplementary file 7 [file Image_5.TIF]
